# Molecular mechanisms of diabetic heart disease: Insights from transcriptomic technologies

**DOI:** 10.1177/14791641231205428

**Published:** 2023-12-20

**Authors:** Marcella Conning-Rowland, Richard M Cubbon

**Affiliations:** Leeds Institute of Cardiovascular and Metabolic Medicine, 573267 University of Leeds, Leeds, UK

**Keywords:** Diabetes, heart, diabetic cardiomyopathy, transcriptomics, RNA-sequencing, gene expression

## Abstract

Over half a billion adults across the world have diabetes mellitus (DM). This has a wide-ranging impact on their health, including more than doubling their risk of major cardiovascular events, in comparison to age-sex matched individuals without DM. Notably, the risk of heart failure is particularly increased, even when coronary artery disease and hypertension are not present. Macro- and micro-vascular complications related to endothelial cell (EC) dysfunction are a systemic feature of DM and can affect the heart. However, it remains unclear to what extent these and other factors underpin myocardial dysfunction and heart failure linked with DM. Use of unbiased ‘omics approaches to profile the molecular environment of the heart offers an opportunity to identify novel drivers of cardiac dysfunction in DM. Multiple transcriptomics studies have characterised the whole myocardium or isolated cardiac ECs. We present a systematic summary of relevant studies, which identifies common themes including alterations in both myocardial fatty acid metabolism and inflammation. These findings prompt further research focussed on these processes to validate potentially causal factors for prioritisation into therapeutic development pipelines.

## Highlights

• Diabetes substantially increases the risk of developing heart failure, even when coronary artery disease and hypertension are not present.
• Factors such as cardiac microvascular disease are thought to contribute to this risk, but we lack strong evidence about the underlying causes.
• This means that we lack treatment specifically targeting heart disease caused by diabetes.
• Transcriptomics offers an unbiased approach to uncover molecular mechanisms and potential therapeutic targets for diabetic heart disease.
• Summarising available transcriptomic data, we show common themes of altered myocardial fatty acid metabolism and inflammation associated with diabetes.

## Introduction

According to the International Diabetes Federation, over half a billion adults across the world now have diabetes mellitus (DM),^1^ 90% of these people have type 2 DM (T2DM). Those with DM are twice as likely to experience major cardiovascular events, such as myocardial infarction, as age-sex matched individuals without DM.^2^ DM particularly increases the risk of heart failure, a primary disease of the myocardium leading to inadequate cardiac output and exercise intolerance. Notably, heart failure often occurs without underling coronary artery disease or hypertension, in which case it has been referred to as ‘diabetic cardiomyopathy’.^3^ This phenomenon clearly illustrates that factors unique to diabetes also cause heart disease, yet our understanding of these remains incomplete.

Vascular dysfunction and disease are a common complications of DM, and affect the entire body; for example, diabetic nephropathy and retinopathy can lead to advanced chronic kidney disease and blindness, respectively.^4^ Organ vasculatures are uniquely specialised to meet the requirements of individual tissues, potentially leading to unique implications of DM for each.^5,6^ Whilst more challenging to study in vivo, the cardiac microvasculature is also susceptible to damage from DM. For example, impaired coronary vasodilation has been demonstrated in people with DM and this correlates with measures of microvascular disease in the kidney.^7^ Alongside this, capillary rarefaction is also observed in the myocardium of people with DM and heart failure compared to controls without DM.^8^ Beyond forming a conduit for blood flow, cardiac endothelial cells (ECs) play other important roles to ensure appropriate cardiac function. For instance, they form a highly selective barrier between the circulation and cardiomyocytes, which contributes to regulated of delivery of fatty acids to cardiomyocytes.^5,9^

**Figure 1.**
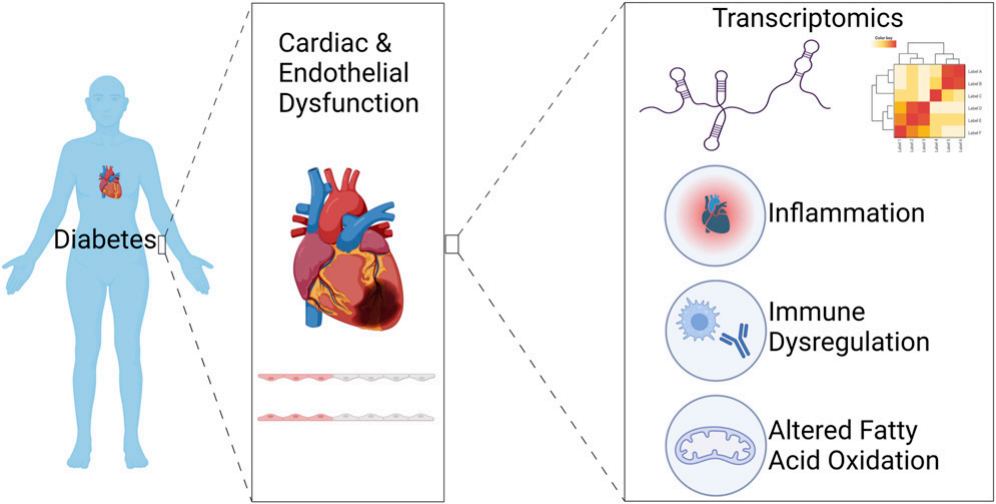
Use of transcriptomics to investigate cardiac and endothelial dysfunction in diabetes has revealed dysregulation in genes associated with fatty acid metabolism and immune/inflammatory responses. Image created with BioRender.com.

Currently, there are no treatments for DM which directly target the heart, potentially reflecting the limited understanding of how DM affects the heart at a molecular level. However, sodium-glucose cotransporter 2 (SGLT2) inhibitors have serendipitously been found to markedly reduce the risk of heart failure in people with DM,^10^ showing that cardiac complications of DM are tractable. SGLT2 is a cotransporter which reabsorbs glucose in the tubular nephron; by inhibiting SGLT2, glucose reabsorption is reduced and is instead excreted in the urine.^11,12^ Despite some evidence indicating direct cardiac effects of SGLT2 inhibitors,^13^ it is likely that they have many indirect effects, such as via altered circulating concentrations of fatty acids and ketones.^14^ Hence, even our understanding of existing therapeutics is limited, further highlighting the need to understand how DM affects the heart and its constituent cells at a molecular level. Recent technological advances mean that this goal is becoming increasingly realistic, for example using an array of unbiased molecular profiling approaches, collectively referred to as ‘omics. This review will predominantly focus on how transcriptomics, for example using RNA sequencing (RNA-seq), can help to define how DM affects the heart and its vasculature, potentially informing the search for novel therapeutic targets (Figure 1).

## Analysis of whole myocardium

Multiple studies have used transcriptomics to quantify the association between DM and altered myocardial gene expression (Table 1); by bringing these results together, general themes of differentially expressed genes (DEGs) are noted. Kesherwani et al demonstrated altered expression of 351 genes in the heart of diabetic Akita versus control mice using RNA-seq.^15^ For example, they found increased expression of Angiopoietin-like-4 (ANGPTL4), phosphoenolpyruvate carboxylase -1 (PCK1), natriuretic peptide type A (NPPA) and pyruvate dehydrogenase kinase 4 (PDK4). The non-targeted approach of RNA-seq allows us to shed light on the function of poorly understood genes/proteins. ANGPTL4 showed the greatest change in RNA expression. The ANGPTL family of proteins interact with Tie receptors, a family of tyrosine kinase receptors mostly expressed on ECs. Modelling predicts that this interaction with the Tie family is altered in inflammatory states, such as is observed in DM.^16^ ANGPTL4 is also associated with regulation of lipid metabolism. It’s production is induced both by fasting and lipids, and it interacts with lipoprotein lipase (LPL) which hydrolyses circulating triglycerides. ANGPTL4 performs distinct roles in different cell types; for example, in adipocytes it promotes fasting-induced degradation of LPL, whilst in macrophages and (cardio)myocytes it is a lipid induced feedback regulator of LPL-mediated lipid uptake.^17^ PCK1 is involved in both gluconeogenesis and lipid synthesis, and promotion of lipid synthesis is associated with development of insulin resistance.^18,19^ PDK4 promotes entry of acetyl-coA from beta-oxidation into the TCA cycle, leading to increased fatty acid oxidation and slowing glycolysis.^20^ NPPA on the other hand, is a clinical biomarker of cardiac stress, further demonstrating that changes in their RNA expression provide credible insights about diabetic heart disease.^21^

**Table 1.** Summary of bulk transcriptomics studies assessing the association of DM on myocardium. PubMed and Google Scholar literature searches were used to select all relevant studies using the search term: “(diabetes OR obesity) AND (myocardium OR cardiac OR heart) AND (RNA-seq OR transcriptomics)”.

Reference	Main message	Diabetic model	Themes of DEGs in DM
Kesherwani, V., Shahshahan, H.R. and Mishra, P.K., 2017^15^	Comparison of RNA-seq and microarray data in DM vs control mice. RNA-seq found 137 DEGs. Microarray found 351 DEGs. 11 genes upregulated in both	Ins2+/- Akita mice	Calcium signalling, protein kinase A signaling, cyclic AMP-mediated signaling, thyroid receptor activation, and estrogen biosynthesis
		Bulk RNA-seq and microarray of cardiac tissue	
Durga Devi, T. et al., 2017^22^	Mechanisms of mortality post myocardial infarction in T2DM. Impaired mitophagy in peri-infarct regions was detected. Release of mitochondrial DNA led to inflammasome and caspase-I hyperactivation & cardiomyocyte cell death	IGFII/LDLR^−/−^ApoB^100/100^ mice fed high fat diet for 12 weeks (+MI induced my ligation of left anterior descending coronary artery)	Heart failure after myocardial infarction in T2DM: Oxidative phosphorylation, mitochondrial dysfunction, TCA cycle II, valine degradation, fatty acid beta-oxidation
		Bulk RNA-seq of cardiac tissue	
Croteau, D. et al., 2021^27^	Effect of SGLT2 inhibition on DM mice. SGLT2 inhibition prevented pathologic cardiac remodelling & induced oxidative phosphorylation and fatty acid metabolism gene sets	Mice fed high fat high sucrose (HFHS) diet for 4 months (+ Ertugliflozin administration in some conditions)	In HFHS hearts, oxidative phosphorylation gene set was downregulated, this was improved by SGLT2 inhibition. Fatty acid metabolism gene set was upregulated in HFHS.
		Bulk RNA-seq of left ventricular tissue	
Wei, C. et al., 2022^28^	Assessing protective role of spermine in diabetic cardiomyopathy	C57Bl/6J mice. DM induced via STZ injection. Additional group administered STZ & spermine	PI3K-Akt signaling pathway, metabolic pathways, ECM receptor interaction, AGE-RAGE signaling pathway in diabetic complications, cell adhesion molecules (CAMs), Ras signalling pathway, HIF-1 signalling pathway, p53 signalling pathway, MAPK signalling pathway, and PPAR signalling pathway
		Bulk RNA-seq of heart tissue	
Liu, F. et al,. 2022^29^	Differentially expressed genes in the atria when atrial fibrillation is induced by hyperglycaemia. MAPK10 identified as key in development of atrial fibrillation	Db/db mice. Assessed at 12, 14, 16 weeks old	Cell adhesion, cellular response to interferon-beta, immune system process, positive regulation of cell migration, ion transport and cellular response to interferon-gamma
		Bulk RNA-seq of left atrial tissue	
Marino, F. et al,. 2023^30^	Comparison cardiac differences in T1DM vs T2DM mouse models. The transcriptomes of the two are markedly different and T1DM displayed worsened cardiomyocyte apoptosis, hypertrophy and fibrosis	T1DM: Induced by high dose STZ, T2DM: Induced by low dose of STZ and 3 weeks high feeding	Top 3 alterations in T1DM vs control: Mitochondrial dysfunction, calcium signalling, cardiac hypertrophy signalling. Top 3 alterations in T2DM vs control: CXCR4 signalling, protein kinase A signalling, glycolysis I. Top 3 alterations in T1DM vsT2DM: Mitochondrial dysfunction, oxidative phosphorylation, CXCR4 signalling
		Bulk RNA-seq of whole heart	
Pan, X. et al,. 2022^31^	UTP14A, DKC1, DDX10, PinX1, ESF1 identified as hub genes responsible for obesity induced cardiac injury	HFD for 8-week	Enriched: Long-term depression, gap junction, sphingolipid signaling pathways
		Microarray analysis of cardiac tissue	
Liu, H. et al,. 2017^26^	VEGF and MAPK signalling may play a role in progressing heart failure in type 2 diabetics	Cardiac tissue samples from T2DM and non-T2DM individuals with heart failure	Focal adhesion, VEGF signalling, MAPK signalling
		Microarray data from E-GEOD-26887 dataset.	
Sárközy, M. et al,. 201^32^	Identification of potential therapeutic targets in the development of cardiac pathologies in non-obese T2DM	Cardiac tissue samples from Goto-Kakizaki rats aged 15 Weeks	Metabolism, signal transduction, receptors & ion channels, membrane and structural proteins, cell growth & differentiation, immune response
		Mircoarray analysis	

Durga Devi *et al* demonstrated that post myocardial infarction, type 2 diabetic mice show altered expression of genes related to metabolic processes such as fatty acid oxidation & oxidative phosphorylation.^22^ Further investigation indicated this could be a result of inhibition of the transcriptional co-activator peroxisome proliferator-activated receptor gamma coactivator (PGC)-1α. Regulated by post-translational modifications from upstream signalling proteins such as AMPK, PGC-1α controls a host of downstream metabolic networks.^23^ There is mixed evidence of PGC-1α’s association with heart disease, therefore further investigation is required.^26^ However, in cardiomyocytes and other highly metabolically active cell types, PGC-1α induces fatty acid oxidation and represses glucose oxidation. It is also responsible for recruiting blood vessels for transport of oxygen/nutrients and has been implicated in vascular dysfunction observed in DM.^24^

RNA-seq can define the full transcriptome, allowing comprehensive detection of splice variants and non-coding RNA, but this is expensive, and analysis can be complex. An alternative approach, predominantly used prior to RNA-seq became widely available, identified differentially expressed genes using microarrays of predefined transcripts.^25^ Microarray data from people with heart failure has demonstrated that Vascular Endothelial Growth Factor (VEGF) is overexpressed in the left ventricles of those with DM.^26^ VEGF predominantly acts on endothelial cells to stimulate angiogenesis and cell survival, along with a variety of metabolic effects, therefore changes in its expression are likely to contribute to EC dysfunction observed in DM.

## Analysis of cardiac EC

Isolation of endothelial cells from a tissue of interest can allow more specific insight into how DM alters their biology (Table 2). EC function is particularly vulnerable to hyperglycaemia as they are highly glycolytic and do not require insulin to induce glucose uptake.^33^ Many studies have demonstrated that DM or DM-mimicking conditions disrupt EC biology, with altered metabolic pathways and oxidative stress leading to reduced viability, sprouting angiogenesis, NO generation and resistance to leakage.^34–37^

**Table 2.** Summary of transcriptomics studies which use isolated ECs assessing the association of DM on myocardium. PubMed and Google Scholar literature searches were used to select all relevant studies using the search terms: “(diabetes OR obesity) AND (myocardium OR cardiac OR heart) AND (RNA-seq OR transcriptomics)AND endothelial”.

Reference	Main message	Diabetic model	Themes of DEGs in DM
Lother, A. et al., 2021^38^	Impact of DM on gene expression and DNA methylation in cardiac cells. 39 DM DEGs in bulk tissue. When sorted into cell types more DEGs were revealed. DNA methylation not altered in DM	Streptozotocin induced diabetic mice	498 DEGs in DM ECs. Contributed equally to DM themes identified in bulk analysis: Fatty acid metabolic process, regulation of leukocyte migration, regulation of leukocyte cell-cell adhesion, inflammatory response, reactive oxygen species metabolic process, positive regulation of immune system process, response to wounding
		RNA-seq of isolated ECs	
Wimmer, A. et al., 2019^39^	Demonstration of blood vessel organoids derived from pluripotent stem cells as models to study DM.	Human iPSC derived organoids transplanted into immunodeficient mice. DM then induced via streptozotocin	GO terms: Collagen biosynthesis & extracellular matrix reorganization. Upregulated genes: ANGPT2, apelin, TNFRSF11B
		RNA-seq of FACs sorted ECs	
Guo, S. et al,. 2019^40^	Ageing, hypertension and DM effect the vasculome of the heart and brain. Large differences were observed between organs and in all conditions the brain vasculome had more DEGs	db/db mice	Upregulated: Rhythmic process, apoptotic process, cell cycle, response to organic cyclic compound, entrainment of circadian clock. Downregulated: Antigen processing and presentation, lymphocyte activation, leukocyte activation
		RNA-seq of cardiac ECs	

Transcriptome analysis of purified cell populations freshly isolated from heart tissue from diabetic mice found 498 genes differentially expressed in ECs that were not apparent in cardiac myocytes, fibroblasts, macrophages, or monocytes. Whilst these genes were uniquely altered in ECs, they contributed to biological processes impacting on other cell types, such as fatty acid metabolism, regulation of leukocyte adhesion/migration, and response to hormones. Notably, in other cell types such as cardiomyocytes, changes to fatty acid metabolism were predominant.^38^

RNA-seq can also be used to phenotype in vitro models of DM, such as organoids. These can be used to gain further insight into how DM alters EC function and test potential therapeutics. Both ECs from vascular organoids derived from human iPSCs exposed to high glucose concentrations, and dermal ECs from human donors with DM, show upregulation of EC matrix structural constituents, growth factor activity and binding, and binding of cell adhesion molecules and integrins.^39^ These data gave confidence that organoids can model the effects of DM on EC and highlighted abnormal Notch signalling as a potential therapeutic target.

## Defining the impact of therapeutic intervention

RNA-seq can also be used to understand the molecular basis of poorly understood therapeutic interventions. For example, the recently developed class of SGLT2 (sodium-glucose linked transporter-2) inhibitors appear to substantially improve cardiac outcomes in people with DM, but the mechanism is unclear. Ertugliflozin is an SGLT2 inhibitor and it prevents cardiac remodelling and improves contractile reserve in mice with high-fat high-sucrose diet induced cardiomyopathy. RNA-seq demonstrated that Ertugliflozin prevents down regulation of the oxidative phosphorylation gene set seen in the untreated high-fat high-sucrose fed mice.^27^ However, it remains unclear whether those transcriptional changes cause, or are simply associated with, the beneficial effects of Ertugliflozin.

HDACs catalyse deacetylation of histones, an epigenetic mechanism for controlling gene transcription and cell signalling, and have been shown to supress GLUT1 transport of glucose. Their inhibition has been suggested may produce favourable outcomes in DM, in particular diabetic kidney disease management.^41–43^ Microarray studies has revealed upregulation of VCAN & CCL2 among other genes in human aortic ECs from people with DM, which was reversed by treatment with a histone deacetylase (HDAC) inhibitor.^44^ Expression of VCAN and CCL2 have been linked with diabetic kidney disease and islet destruction respectively, therefore this evidence suggests their effect on the diabetic vascular should be further investigated.^45,46^

## Emerging technological advances: scRNA-seq

Bulk RNA-seq provides data on averaged gene expression from all cells in a sample. Single cell RNA-seq (scRNA-seq) provides gene expression data for all of those individual cells, with modern experiments encompassing hundreds of thousands or even millions of cells (Table 3).^47^ scRNA-seq of heart and aorta from atherosclerosis prone LDL receptor knockout mice has demonstrated that ECs are heterogeneous and have distinct sub-clusters even within the same organs.^48^ Following high fat diet feeding for 12 weeks, scRNA-seq of isolated purified ECs from the murine heart demonstrated different responses to the diabetogenic diet in 8 different EC clusters; notably, some populations were unaltered whereas others populations disappeared, and endothelial-mesenchymal transition was increased. A cluster of EC defined by intense expression of lipid metabolism genes was 3-fold more abundant in mice exposed to the diabetogenic diet, representing nearly half of all EC. Notably, the expression of lipid metabolising genes did not differ in this cluster in diabetic versus control mice, suggesting a difference in the abundance of the cells, rather than their function.

**Table 3.** Summary of scRNA-seq studies which use ECs assessing the association of DM on myocardium. PubMed and Google Scholar literature searches were used to select all relevant studies using the search terms: “(diabetes OR obesity) AND (myocardium OR cardiac OR heart) AND (scRNA-seq OR single cell)AND endothelial”.

Reference	Main message	Diabetic model	Themes of DEGs in DM
Bondareva, *et al.,* 2022^49^	scRNA profiling of 375000 ECs across seven organs, in obese or control conditions, obesity dysregules lipid handling, metabolism, inflammatory signalling, in organ specific patterns. Dietary intervention partially mitigated effects of sustained obesity	Mice fed western diet for 3-month. ECs isolated by FACs sorting	Cardiac EC clusters: KLF transcription factor genes induced (vascular inflammation and shear stress)
			Cardiac arterial ECs: Enrichment of genes associated with shear stress, atherosclerosis, ECM-receptor interaction, leukocyte transendothelial migration
			High AP1 transcription factor cardiac EC cluster: Induction in inflammatory pathways
Zhao, G. *et al.,* 2021^48^	scRNA-seq of ECs to investigate DM aggravated atherogenesis. 8 EC clusters identified, 3 clusters showed markers of transition to mesenchymal cells	EC enriched single cells from mouse heart & aorta following 12 weeks high fat diet with cholesterol	Cluster of ECs defined by high expression of lipid metabolism increased in abundancy in DM.
Li, W. *et al.*, 2023^50^	Focus on myocardial fibrosis in diabetic cardiomyopathy. Looked into intercellular communication	Mice with 6 weeks HFD feeding and STZ injection	Pdgfb and Pdgfd transcripts were upregulated. Top 10 upregulated genes in ECs: Adamts9, Angptl4, Btnl9, Herc6, Ifi44, Oas2, Oasl2, Pdk4, Rnf213, Txnip
Dona, M. *et al.*, 2023^51^	Western diet induces inflammatory signature in non-myocyte populations of cardiac tissues. Deletion of mineralocorticoid receptor in smooth muscle cells reduced this effect	Mice fed western diet for 16 weeks scRNA-seq performed on cells isolated from left ventricle	9 EC populations. Upregulated in EC populations: Antigen processing & presentation, leukocyte activation

scRNA seq also demonstrates that ECs from diverse organs do not respond in the same way a diabetogenic stimulus. Cardiac ECs isolated from mice fed a high-fat diet for 3 months showed increased expression of inflammatory mediators, including the several Krüppel-like transcription factors (KLF), in contrast with other organs. Additionally, nerve growth factors, such as Brain Derived Neurotrophic Factor (BDNF), were induced in cardiac ECs of obese mice.^49^ The authors identified 11 distinct EC clusters in the heart, the proportions of which, and gene expression profiles within, were also differentially influenced by high-fat diet; for example, leukocyte trans-endothelial migration genes were altered in arterial ECs, whilst extracellular matrix genes were increased in capillary ECs. These data emphasise that even within an organ, DM-inducing stimuli have heterogeneous effects. Moreover, common in vitro models of EC responses to diabetogenic factors (e.g. using human umbilical vein or artery EC) are likely to diverge substantially from in vivo cardiac EC responses.

## Common biological themes emerging from transcriptomic studies

General themes of altered biological processes such as metabolism and inflammation have emerged across the previously discussed studies. However, it is worth noting that these responses may not be unique to DM, and rather represent a broader pathological response. For example, studies investigating aortic stenosis have also demonstrated dysregulated fatty acid oxidation in cardiomyocytes and cardiac ECs.^52,53^ Irrespective of this, they may still represent interesting therapeutic opportunities.

### Fatty acid metabolism

A consistent observation noted in the studies presented earlier is the effect of DM on cardiac fatty acid metabolism. Kesherwani *et al* showed upregulation of PDK4 in the myocardium of diabetic mice.^15^ PDK4 is a mitochondrial protein which controls the flux of acetyl-CoA entering the TCA cycle from fatty acid oxidation or glycolysis. This occurs through inhibition of glucose utilisation in the TCA cycle, therefore the observed increase in PDK4 may translate to increased fatty acid metabolism.^20^ PDK4 was also differentially expressed in the ECs isolated from the left ventricles of diabetic mice investigated by Lother *et* al.^38^ In fact, all cell types included in this investigation showed enrichment of genes related to fatty acid metabolism. In ECs, 6 of the 10 genes associated with the gene ontology term “Fatty acid beta-oxidation using acyl-CoA dehydrogenase” showed differential expression. This included acyl Co-A dehydrogenase (ACAD) in it’s medium, long and very long versions, variants with differing preferred chain lengths of fatty acyl-CoA substrates. These mitochondrial flavoenzymes catalyse the rate limiting desaturation of acyl-CoA esters required for fatty acid β-oxidation.^54^ The alteration of multiple members of the ACAD family strongly supports the notion that DM alters the metabolism of fatty acids.

Both these studies used murine models of DM more closely replicating Type 1 DM (T1DM) (Akita and Streptozotocin (STZ) models). However, Durga Devi *et al* used mice which overexpressed IGFII in pancreatic beta cells, crossed with hypercholesterolaemic LDLR^-/−^ApoB^100/100^ mice, with high fat diet feeding for 12 weeks, to model T2DM; myocardial infarction was then surgically induced by ligating a coronary artery.^22^ Left ventricular tissue from these mice also showed differential expression of ACAD enzymes - the short, medium and very long versions, supporting that with diverse forms of DM have altered cardiac fatty acid oxidation. In this study, gene sets relating to oxidative phosphorylation, mitochondrial dysfunction, leucine, valine, and tryptophan degradation as well as fatty acid oxidation were also altered. However, it should be noted that the tissue used in this study came from infarct and peri-infarct regions of tissue, which adds further complexity to their data.

Croteau *et al* also used a mouse model representative of T2DM (high-fat, high-sucrose feeding), when investigating the effect of sodium-glucose linked transporter 2 inhibition on myocardium. This work also strongly supports findings of altered fatty acid metabolism gene sets in diabetic tissue. In the left ventricle of the diabetic mice, increase in fatty acid metabolism and decrease in oxidative phosphorylation gene sets was observed. Fatty acid oxidation and oxidative phosphorylation pathways are intrinsically linked, with fatty acid oxidation supplying reducing equivalents to oxidative phosphorylation. Again, the ACAD enzymes (short, medium, long, and very long), were differentially expressed alongside 25 other fatty acid oxidation related genes. There was an even greater number (*n*=104) of differentially expressed genes related to oxidative phosphorylation, including 6 components of the Ubiquinol-cytochrome c reductase complex.^27^ This complex forms part of the mitochondrial respiratory chain, and has previously been demonstrated as displaying decreased activity in multiple tissues from STZ induced diabetic rats.^55^ These same components of this complex were also all differentially expressed in the previously discussed model using infarct/peri-infarct tissue in diabetic tissue.

Alongside this RNA-seq based evidence that DM alters fatty acid metabolism, proteomics work has led to similar conclusions, providing an alternate form of validation. A study measuring protein expression in the left ventricles of STZ-induced diabetic rats, also demonstrated an increase in proteins involved in fatty acid metabolism. Of particular note, is that multiple isoforms of ACAD long chain specific, showed increases of 47%–109% in STZ-diabetic hearts.^56^

### Immune dysregulation and inflammation

DM is well known to affect the immune system, and targeting inflammation is an increasing focus of investigation for novel therapeutics^57^ This is reflected in the range of immune function related hits observed in the RNA-seq experiments described above. Durga Devi *et al* demonstrated that inflammasome related genes (e.g. Aim2, Nlrc4) were upregulated in peri-infarct and infarct regions of the left ventricle of type 2 diabetic mice compared to controls.^22^ Inflammasomes are protein complexes which induce inflammation in response to threats such as foreign microbes; upregulation of these two proteins may contribute to inflammation in the left ventricle regions.^58^ Increased inflammation in these diabetic mice was confirmed by follow up experiments which quantified circulating IL-18, an inflammatory mediator, and found it to be significantly increased even in type 2 diabetic mice which did not undergo myocardial infarction.

Similarly, Lother *et al* found multiple genes associated with antigen presentation had altered expression in cardiac EC isolated from diabetic mice, namely B2m, H2-K1 and Tap2. B2m is a subunit of major histocompatibility complex (MHC) class I and Tap 2 forms a complex with Tap1 which attaches to MHC class 1 molecules, while H2-K1 enables the antigen presentation process. Altered expression of multiple antigen presentation genes in the absence of stimuli, suggests dysregulation of the immune response, potentially contributing to inappropriate inflammation.

One of the top upregulated genes in myocardium of diabetic mice, found by Kesherwani, *et al* was CD207, also known as langerin which also regulates immune responses.^15^ Langerin is an antigen uptake receptor most often expressed by Langerhans cells, which stimulates T cell responses.^59^ Presumably an increase in langerin would contribute to increased T cell activation and stimulation of immune responses. However, Langerhans cells most often reside in the oral mucosa and the skin epidermis, therefore the relevance of this increase in myocardial langerin is unclear.

Proteomics studies also demonstrate dysregulation of the immune system and inflammation in DM. The HOMAGE trial measured protein biomarkers in the plasma of participants with and without DM.^60^ Enriched GO processes found in this study included immune response, immune system process and inflammatory response, supporting inflammatory perturbation in DM mellitus. Interestingly, TNFRSF11B is one of the significantly differently expressed protein biomarkers in people with DM. The TNFRSF11B gene was also found to be upregulated in iPSC-derived EC cultured in high glucose concentrations in a vascular organoid model of diabetic vascular disease.^24^ This gene is involved in infiltration of T cells in colorectal cancer and may have similar relevance in diabetic cardiovascular disease.^61^

## Translation to therapy

Whilst ‘omics investigations provide a broad insight into the physiological state of the diabetic heart, a major goal of these studies is to identify targets for prevention and treatment of heart disease in people with DM. One example of how an improved understanding of ‘omics approaches have led to improved treatment outcomes comes from proprotein convertase subtilisin/kexin type 9 (PCSK9). PCSK9 is an essential regulator of lipid metabolism and mutations in the PCSK9 gene are linked to autosomal dominant hypercholesterolemia, a risk factor for cardiovascular disease.^62^ Genome wide association studies (GWAS) demonstrated that PCSK9 gain of function mutations are associated with increased risk of developing coronary heart disease.^63,64^ As a result of such data, PCSK9 inhibitors were developed and were shown in clinical trials to reduce major cardiovascular events even in people receiving statins.^65^ If this approach could be emulated using targets identified by other ‘omics approaches studying diabetic heart disease, it is possible that improved treatments could be developed.

Once genes associated with cardiac disease in people with DM have been identified by RNA-seq studies, further research is required to understand if it is an appropriate therapeutic target. Mendelian randomisation (MR) has proven a powerful approach to infer causal associations using genetic data, prior to therapeutic studies.^66^ MR is not applicable to non-genetic data, but alternate statistical approaches such as mediation analysis^67^ also allow causal inference testing to validate targets for ongoing therapeutic targeting studies. Equally, manipulating identified targets using genetic or pharmacological approaches applied to in vitro or in vivo models of diabetic heart disease is an essential part of validating novel therapeutic targets.

‘Omics technologies like RNA-seq can also help to better understand the mechanisms by which current therapeutics work, as highlighted earlier in the discussion of SGLT2 inhibitors. Such data not only aid our understanding of how agents might work, but could also help to refine their use or develop novel therapeutic targets.

## Conclusion

RNA-seq can provide broad insights into how DM impacts on the heart. Although research specific to cardiac ECs in vivo is more limited, there is emerging evidence that their transcriptome is also substantially altered by DM. In both cardiac ECs and whole myocardium, genes related to regulation of fatty acid metabolism and inflammation are frequently reported to be altered in diverse models of DM. However, some of these observations may not be specific to DM, instead denoting broader pathological responses. Whilst some RNA-seq hits discussed above have been taken forward and investigated in relation to therapeutics, there remain many unexplored avenues which could be investigated to assess their translational potential. However, larger studies involving myocardium and isolated EC from people with DM are likely to be required to successfully underpin translational studies. As RNA-seq, in both bulk and single cell formats, becomes cheaper and data sharing initiatives emerge, it is likely that such data will become a powerful tool to understand and address diabetic heart disease.

## Appendix

### Nomenclature

ACAD: acyl Co-A dehydrogenase
ANGPTL4: Angiopoietin-like-4
BDNF: Brain Derived Neurotrophic Factor
EC: Endothelial Cell
CAM: Cell Adhesion Molecule
DEG: Differentially Expressed Gene
DM: Diabetes Mellitus
GWAS: Genome Wide Association Studies
HDAC: Histone Deacetylase
HFHS: High Fat High Sucrose
KLF: Krüppel-like Transcription Factors
LPL: Lipoprotein Lipase
MHC: Major Histocompatibility Complex
MR: Mendelian Randomisation
NPPA: Natriuretic Peptide Type A
PCK1: Phosphoenolpyruvate Carboxylase -1
PCSK9: Proprotein Convertase Subtilisin/Kexin Type 9
PDK4: Pyruvate Dehydrogenase Kinase 4
PGC: Proliferator-activated receptor Gamma Coactivator
RNA-seq: RNA-sequencing
scRNA-seq: Single Cell RNA-sequencing
SGLT2: Sodium-Glucose Cotransporter 2
STZ: Streptozotocin
T2DM: Type 2 Diabetes Mellitus
VEGF: Vascular Endothelial Growth Factor
